# In vitro biotransformation of pyrrolizidine alkaloids in different species: part II—identification and quantitative assessment of the metabolite profile of six structurally different pyrrolizidine alkaloids

**DOI:** 10.1007/s00204-020-02853-9

**Published:** 2020-09-03

**Authors:** Ina Geburek, Dieter Schrenk, Anja These

**Affiliations:** 1 Department Safety in the Food Chain, German Federal Institute for Risk Assessment, Max-Dohrn-Straße 8-10, 10589 Berlin, Germany; 2 Food Chemistry and Toxicology, University of Kaiserslautern, Erwin-Schrödinger-Straße 52, 67663 Kaiserslautern, Germany

**Keywords:** Pyrrolizidine alkaloids, In vitro metabolism, Metabolite profile, Biotransformation, Mass spectrometry

## Abstract

Pyrrolizidine alkaloids (PA) exert their toxic effects only after bioactivation. Although their toxicity has already been studied and metabolic pathways including important metabolites were described, the quantification of the latter revealed a large unknown portion of the metabolized PA. In this study, the qualitative and quantitative metabolite profiles of structurally different PAs in rat and human liver microsomes were investigated. Between five metabolites for europine and up to 48 metabolites for lasiocarpine were detected. Proposals for the chemical structure of each metabolite were derived based on fragmentation patterns using high-resolution mass spectrometry. The metabolite profiles of the diester PAs showed a relatively good agreement between both species. The metabolic reactions were summarized into three groups: dehydrogenation, oxygenation, and shortening of necic acid(s). While dehydrogenation of the necine base is considered as bioactivation, both other routes are considered as detoxification steps. The most abundant changes found for open chained diesters were dealkylations, while the major metabolic pathway for cyclic diesters was oxygenation especially at the nitrogen atom. In addition, all diester PAs formed several dehydrogenation products, via the insertion of a second double bond in the necine base, including the formation of glutathione conjugates. In rat liver microsomes, all investigated PAs formed dehydropyrrolizidine metabolites with the highest amount formed by lasiocarpine, whereas in human liver microsomes, these metabolites could only be detected for diesters. Our findings demonstrate that an extensive analysis of PA metabolism can provide the basis for a better understanding of PA toxicity and support future risk assessment.

**Electronic supplementary material:**

The online version of this article (10.1007/s00204-020-02853-9) contains supplementary material, which is available to authorized users.

## Introduction

Pyrrolizidine alkaloids (PA) are a large group of phytotoxins which are found worldwide in about 3% of all flowering plants (Smith and Culvenor 1981). Due to their high structural diversity, over 660 different PAs and PA *N oxides* have been identified (Fu et al. 2002). PAs can be classified into monoesters, open chained diesters, and cyclic diesters based on the degree of esterification of the pyrrolizidine base diol with necic acids. Some of the PAs are hepatotoxic and responsible for cases of poisoning in humans and livestock via cytochrome P450 enzyme (CYP)-mediated metabolic activation (Mohabbat et al. 1976; Molyneux et al. 2011; Tandon et al. 1976). The biotransformation rate of PAs, more precisely the extent of metabolic activation versus detoxification, may significantly influence their toxicity. In addition, differences in relative potency may also be influenced by other factors such as toxicokinetics, i.e., cellular uptake and efflux of the parent PA (Hessel et al. 2014; Ning et al. 2019; Tu et al. 2013, 2014) and half-live and reactivity of the electrophilic metabolites.

It has been proposed that metabolic routes like hydrolysis of the ester bonds leading to the retronecine- or heliotridine-type necine base as well as the conversion of the tertiary necine base into its corresponding *N*-oxide represent major detoxification steps (Fu et al. 2004a). The 1,2-unsaturated ring structure of the necine base seems to be the precondition for PAs to exert their toxicity (Mattocks 1968; Ruan et al. 2014a). The oxidative conversion of 1,2-unsaturated PAs by hepatic enzymes leads to reactive intermediates like dehydropyrrolizidine derivatives (pyrrolic derivatives) which are considered to cause the toxic effects (Miranda et al. 1991; White et al. 1973). These unstable electrophiles are able to react with nucleophilic macromolecules like proteins or DNA (Fu et al. 2004a; He et al. 2019; Ruan et al. 2014b; Zhao et al. 2012). The resulting genotoxic effects such as DNA adduct formation, micronuclei generation, or chromosomal abberations are well studied (Allemang et al. 2018; Chen et al. 2010; Müller et al. 1992; Ribeiro et al. 1993; Wang et al. 2005; Yang et al. 2001). Xia et al. investigated whether secondary pyrrolic metabolites can bind to calf thymus DNA and to cellular DNA in HepG2 cells, resulting in the formation of ( ±)-6, 7-dihydro-7-hydroxy-1-hydroxymethyl-5H-pyrrolizidine (DHP)-DNA adducts. The authors found that many secondary pyrrolic metabolites are DNA reactive and can form DHP-DNA adducts, and suggested that secondary pyrrolic metabolites play a vital role in the initiation of PA-induced liver tumors (Xia et al. 2018). Xia et al. also reported that DHP conjugated to GSH is able to form DNA adducts, although conjugation to GSH is considered as a detoxification pathway (Xia et al. 2015). Therefore, we investigated in our study whether still unknown pyrrolic PA metabolites or GSH conjugates are formed.

In the development of pharmaceuticals, drug‐induced liver injury due to reactive metabolites is the most frequent cause of drug failures in clinical trials. A common practice in the testing of drug candidates includes assessing the formation of reactive metabolites using mass spectrometric screening approaches with soft and hard nucleophiles in liver microsomes (Jian et al. 2012; Ramirez-Molina and Burton 2009; Rousu et al. 2009). Glutathione (GSH) acts as trapping agent for those reactive metabolites as, for instance, pyrrolic metabolites. Covalent GSH conjugates with dehydropyrrolizidine were identified as metabolites of senecionine and several other PAs, and considered as an indirect confirmation of the bioactivation pathway (Chen et al. 2016; Geburek et al. 2020; Huan et al. 1998b; Lin et al. 1998; Ramsdell and Buhler 1987; Reed et al. 1992). A study by Yan et al. investigated 22 PAs as potential targets in GSH metabolism, and they identified glutathione S-transferase A1 (GSTA1) and glutathione peroxidase 1 (GPX1) as protein targets (Yan et al. 2016).

Although much research has focused on the identification of PA metabolites (Buhler and Kedzierski 1986; Fashe et al. 2014, 2015; Fu et al. 2004a), their quantification revealed that a large portion is still unknown. Furthermore, GSH-DHP conjugates like 7,9-diglutathionyl-6,7-dihydro-1-hydroymethyl-5H-pyrrolizidine (diGSH-DHP), and 9-glutathionyl-6,7-dihydro-1-hydroxymethyl-5H-pyrrolizidine (monoGSH-DHP) suggested as biomarkers for in vitro metabolic activation were not detectable when CYP content is low—for instance when using S9 as metabolic system instead of microsomes, or were only detectable after long-term incubation (Ebmeyer et al. 2019; Geburek et al. 2020; Kolrep et al. 2018).

In the previous studies, we demonstrated that liver microsomes from species considered to be sensitive to PAs showed a lower metabolic rate than liver microsomes from species considered to be more resistant (Kolrep et al. 2018). This observation could be explained by the fact that the observed high overall rate of microsomal biotransformation in non-susceptible species mainly represents a detoxification. In contrast, metabolic activation leading to reactive metabolites, although low in quantitative terms, may be crucial in microsomes from susceptible species.

In this study, the metabolite profile of structurally different PAs formed by rat (RLM) and human (HLM) liver microsomes was comprehensively identified and structurally characterized. Furthermore, we used protocols established for drug candidate testing and conducted a broad range of reactive metabolite “trapping” experiments to clarify if still unknown GSH or comparable conjugates are formed during PA bioactivation.

**Fig. 1:**
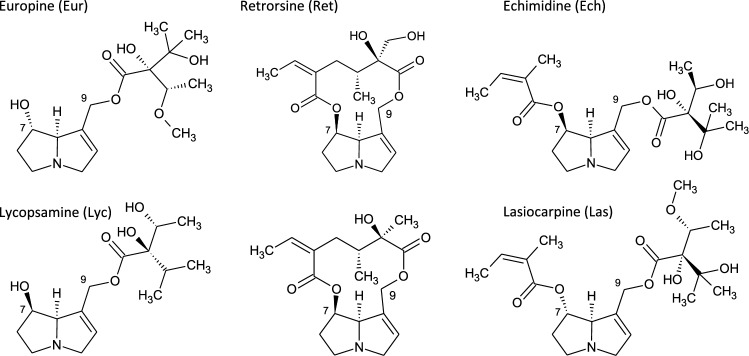
Molecular structures of six investigated pyrrolizidine alkaloids (PAs). Different 1, 2-unsaturated necine base structure types were involved like the heliotridine type (C7S; europine, lasiocarpine) and the retronecine type (C7R; echimidine, lycopsamine, retrorsine, and senecionine). Additionally, different ester types like monoesters and open chained diesters and cyclic diesters were studied

Combined approaches were applied for metabolite identification: (1) screening techniques like neutral-loss and precursor ion scans applicable to search for typical structural units like the retronecine/heliotridine core structure, (2) typical phase I and phase II transformation steps were checked by so-called expected workflows, and (3) a software-assisted chromatographic peak detection was applied enabling for an unknown-search. For an unambiguous confirmation of screened candidates as PA metabolites, highly resolved product ion scans were acquired and the fragments were compared with those of the reference substances and literature data (Fashe et al. 2014, 2015; Ma et al. 2015). We expanded the recently published method for the detection and quantification of the metabolic transformation and GSH conjugate formation of PAs in microsomes from different species (Geburek et al. 2020; Kolrep et al. 2018) to elucidate the metabolite profile of six PAs (Fig. 1). Metabolites were generated by human and rat liver microsomes and analyzed by Ultra-High-Performance Liquid Chromatography (UHPLC) in combination with high-resolution mass spectrometry (HRMS) to demonstrate differences in metabolism in relation to species and PA structure.

## Materials and methods

### Chemicals and reagents

Lasiocarpine (Las) and lasiocarpine *N*-oxide were ordered from Cfm Oskar Tropitzsch (Marktredwitz, Germany). Lycopsamine (Lyc), lycopsamine *N*-oxide, europine (Eur), europine *N*-oxide, senecionine (Sen), senecionine *N*-oxide, echimidine (Ech), echimidine *N*-oxide, intermedine, intermedine *N*-oxide, jacobine, jacobine *N*-oxide, heliosupine, heliosupine *N*-oxide, retrorsine *N*-oxide, retronecine, and senkirkine were obtained from Phytolab (Vestenbergsgreuth, Germany). Retrorsine (Ret) was delivered by AppliChem (Darmstadt, Germany). Methanol (MeOH, LC–MS grade) and water (H_2_O, LC–MS grade) were purchased from Merck KGA (Darmstadt, Germany). 7,9-Diglutathionyl-6,7-dihydro-1-hydroymethyl-5H-pyrrolizidine was obtained from ASCA (Berlin, Germany). Potassium cyanide (KCN), isotopically labeled potassium cyanide (KCX, K^13^C^15^N), glutathione and isotopically labeled glutathione (GSX, ^13^C_2_^15^N-GSH), and all other chemicals and co-factors were purchased from Sigma-Aldrich (Steinheim, Germany). All chemicals were obtained in analytical grade, if available. Human liver microsomes (mixed gender from 50 donors; protein concentration 20 mg/mL; cytochrome P450 content 310 pmol/mg protein) and rat liver microsomes (male Sprague–Dawley rats; protein concentration 20 mg/mL; cytochrome P450 content 700 pmol/mg protein) were obtained from Corning (Corning, USA).

### Incubation of PAs with liver microsomes

The in vitro phase I and II metabolite profile was obtained via incubation with HLM or RLM. All incubation mixtures were prepared on ice in a final volume of 250 µL tris buffer (pH 7.5, 50 mM) containing HLM or RLM (1 mg protein/mL), 15 µM of the respective PA, 33 mM potassium chloride, 8 mM magnesium chloride, 1 mM nicotinamide adenine dinucleotide phosphate (NADPH), 5 mM glucose-6-phosphate, and 0.5 U/mL glucose-6-phosphate dehydrogenase. Glutathione at a final concentration of 5 mM was added for simulating phase II metabolism and formation of GSH conjugates.

Screening experiments for reactive metabolites were performed with lasiocarpine and RLM in the presence of nucleophilic trapping agents, such as GSH, KCN, or N-acetylcysteine (NAC). Isotope-labelled trapping agents, if available, were used in parallel (Argoti et al. 2005; Deng et al. 2018; Dieckhaus et al. 2005; Jian et al. 2012; Yan and Caldwell 2004). The same incubation mixture as described above was used, with the following modifications: 4 mg/mL RLM either combined with 2 mM GSH and 2 mM GSX or 1 mM KCN and 1 mM KCX. Furthermore, an approach with 5 mM NAC was prepared, which additionally contained 4 mg/mL rat liver cytosol, 8 mM ATP, and 0.8 mM coenzyme A.

All incubations were performed at 37 °C for 60 min at 400 rpm. At 0, 5, 10, 30, and 60 min, an aliquot was removed from each incubation tube and the reaction was stopped by adding ice-cold methanol containing 1% ammonium formate and vortexing. Several experiments were conducted for optimization and validation of the incubation procedure (Geburek et al. 2020). The repeatability was in independent experiments shown to be satisfactory. Three controls were analyzed for each compound. The first control included the PA, water, and Tris buffer, only. This control sample was included to check whether PAs are stable in this buffer. The second control consisted of the buffer and the co-factors for phase I + II metabolism, and microsomes were not added. This control was used to check whether PAs are also transformed enzyme-independently by the cofactor only. Stability of the PA in this buffer confirmed the enzyme dependence of PA transformation in the incubation mixture. The third control was composed of buffer and microsomes, and no cofactor was added. This control was conducted to determine whether PAs are metabolized by NADPH-independent microsomal enzymes. Stability of PAs in this control indicated that PAs were only metabolized by the NADPH-dependent enzymes in the incubation mixture. In addition, a solvent control was also performed, which only contained the incubation mixture and methanol instead of the PA. All controls were performed by sampling at the beginning and at the end of incubation. Thus, a misidentification of substances non-specifically formed by the microsomes or chemicals could be excluded. All samples were stored at − 80 °C until further processing. The samples were centrifuged at 14,000 × *g* at 4 °C for 10 min and the supernatant was diluted with 5% methanol prior to the measurement. For the quantification of unreacted PA substrate and formed metabolites, an 11-point matrix-matched calibration curve (0.001 µM; 0.005 µM; 0.01 µM; 0.05 µM; 0.1 µM; 0.5 µM; 1 µM; 5 µM; 10 µM; 15 µM; 20 µM) was prepared for each PA. The calibration levels were handled in the same way as the incubation samples, except that they were stopped immediately and not incubated. All experiments were performed in duplicate.

### Liquid chromatographic analysis

All measurements were conducted on an UltiMate 3000 (Thermo Fisher Scientific, Waltham, USA) Ultra-High-Performance Liquid Chromatography (UHPLC) system. Chromatographic reversed-phase (RP) separation with 5 µL injection volume was performed on a C18 Hypersil Gold column (150 mm × 2.1 mm; 1.9 μm particle size) with guard column (Thermo Fisher Scientific, Waltham, USA) at a flow rate of 0.3 mL/min and with a column temperature of 40 °C. The binary mobile phase was composed of water as mobile phase A and methanol as mobile phase B, both containing 0.1% formic acid and 5 mM ammonium formate. The gradient conditions were as follows: 0–0.5 min A: 95% / B: 5%, 7.0 min A: 50%/B: 50%, 7.5 min A: 20% / B: 80%, 7.6 min A: 0% / B: 100%, 10.1–15 min A: 95% / B: 5%.

### Mass spectrometry

High-resolution mass spectrometry experiments were carried out on a Q Exactive Focus Orbitrap system (Thermo Fisher Scientific, Waltham, USA). PAs and their metabolites were ionized via electrospray ionization in the positive mode. To identify candidates as PA metabolites high-resolution product, ion scans (ddMS2) with collision energies of 15–35 eV were acquired for each metabolite. Variable data-independent acquisition was applied for quantitative analysis. Here, full-scan data were recorded for the mass range of *m/z* 100–1,500 using a resolution of 70,000. In parallel, a fragmentation mode is combined to generate MS2 data for selected mass range windows (here, m/z: 100–500; 500–1,000; 1,000–1,500), which were acquired with a resolution of 17,500.

Reactive metabolite screenings were analyzed in positive and negative ion mode using the Agilent 6495 Triple Quadrupole system combined with an Agilent 1290 Infinity II LC System (Agilent Technologies; Waldbronn, Germany). The cyanide conjugates were screened in the positive mode via a constant neutral-loss scan (NL) of 27 and 29 Da. The GSH conjugates were screened in the positive mode as follows: via an NL of 129 and 132 Da, NL of 147 and 150 Da, and NL of 307 and 310 Da, via precursor ion scan in the positive mode of m/z 118, m/z 256 and 259, and m/z 274 and 277. GSH conjugates were additionally investigated in the negative ion mode by a precursor ion scan of m/z 143 and 146, m/z 254 and 257, and m/z 272 and 275. NAC conjugates were analyzed in the positive mode via NL of 163 Da and via precursor ion scan of m/z 164 in the positive mode and m/z 162 in the negative mode. For all screening experiments, a mass range of m/z 300–1,300 was analyzed, while a collision energy of 28 eV was applied.

### Identification and quantification of metabolites

Targeted and untargeted metabolomic workflows were performed using the Compound Discoverer software (Thermo Fisher Scientific, Waltham, USA), which enables the recognition of chromatographic peaks which are present in the incubation samples but absent in the blank or control samples. The search was applied with wide-set filters and a minimum of limitations concerning the peak recognition algorithm. All samples were analyzed with both, an expected and an unexpected workflow for a maximum number of three combined reactions.

To confirm a candidate as an identified metabolite several criteria had to be fulfilled: (1) the deviation of the measured accurate mass and the sum formula derived for the metabolites had to be below 1 ppm including the necessity of a matching isotopic pattern, (2) the recorded highly resolved product ion scan had to contain fragments as well as fragmentation patterns known to be specific for the PAs and for the metabolites already described in the literature, and (3) no metabolite was allowed to be present at the start of incubation and its concentration had to increase over time.

Furthermore, for each identified metabolite, proposals for chemical structures were suggested based on mass spectrometric fragmentation. In some cases, the metabolic transformation could only be assigned to a certain molecule part within the PA. Due to the positively charged nitrogen atom of the necine base (refer to Fig. 1), this necine base part can be detected by very specific fragments formed during fragmentation in the collision cell of the mass spectrometer. These fragments allow a clear statement whether the metabolic reaction took place in the necine base or at the necic acid. As no metabolites were available as references standard, a semi-quantitative approach was used by assuming the same mass spectrometric response for the metabolites as for the PA educts, except the corresponding *N* oxides and the GSH conjugates, which were quantified with the available standards.

To evaluate the differences in the mass spectrometric response between PAs and their metabolites, we analyzed a selection of 18 different analytes, which represent different types of transformations such as *O*-demethylation (lasiocarpine and heliosupine), *N*-demethylation (senkirkine and senecionine), carbon oxygenation and epoxidation (senecionine versus retrorsine and jacobine or *N*-oxidation (PA vs. PANO), and ester cleavage to retronecine. The highest MS signal was recorded for senkirkine and the MS response of all other analytes was calculated in relation to the signal of senkirkine. The order of the analytes according to their MS response was as follows: senkirkine (100%), heliosupine *N*-oxide (98%), intermedine *N*-oxide (96%), heliosupine (82%), lasiocarpine *N*-oxide (74%), lasiocarpine (74%), echimidine *N*-oxide (69%), senecionine *N*-oxide (64%), intermedine (63%), europine *N*-oxide (57%), europine (49%), senecionine (47%), echimidine (43%), jacobine (43%), jacobine *N*-oxide (41%), retrorsine (40%), retrorsine *N*-oxide (25%), and retronecine (17%). The highest variation in the ESI MS response was found to be 5.8-fold. These results reveal that using parent PA response to calibrate the response of metabolites can lead to incorrect quantification, but due to the absence of alternatives, the semi-quantitative approach was used to estimate metabolite concentrations.

### Software

The depletion of PAs and the formation of metabolites were detected and quantified with Tracefinder (Thermo Fisher Scientific, Waltham, USA). All ddMS2 data were evaluated with Xcalibur (Thermo Fisher Scientific, Waltham, USA). Additionally, Compound Discoverer was used to screen for further unknown metabolites with both an expected and an unexpected workflow.

## Results

**Fig. 2:**
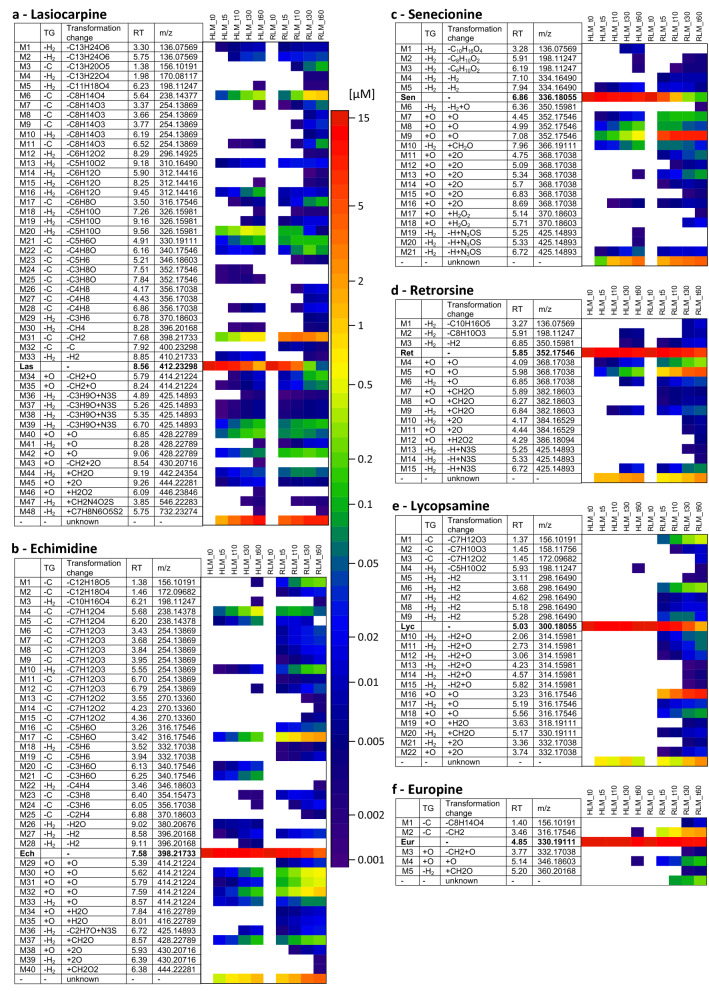
List of all identified metabolites of lasiocarpine (**a**), echimidine (**b**), senecionine (**c**), retrorsine (**d**), lycopsamine (**e**), and europine (**f**). The metabolites of each PA are arranged according to their molecular weight (m/z). The transformations are grouped into three transformation groups (TG): −H_2_: dehydrogenation group (the insertion of an additional double bond); −C: shortening of alkyl chain; + O: oxygenation reactions. The additionally stated transformation change represents the absolute change of the elemental composition. *RT* Retention time in HPLC. *HLM* human liver microsomes, *RLM* rat liver microsomes. The results are presented separately for each point in time (t0, t5, t10, t30, t60 min) in a heat map showing the levels of the metabolites in a logarithmic scale (µM)

The aim of the current study was to elucidate the metabolite profile of six structurally different PAs. For this purpose, each PA was incubated with human and rat liver microsomal preparations with and without glutathione, and samples were taken at different points in time to map the time course of metabolite formation. None of the metabolites was detected in the controls, indicating that all of them were formed in a CYP- and NADPH-dependent manner. In addition, PA stability was confirmed by respective controls in the absence of metabolizing enzymes. The number and amount of all metabolites of all PAs tended to increase over time. The recovery decreased significantly for lasiocarpine from 90% (*t* = 5 min) to 30% after 60 min incubation and moderately from 95–70% for all other PAs (Fig. 2). Observed biotransformations included shortening of necic acids by demethylation and loss of alkyl groups as well as introduction of oxygen, e.g., by hydroxylation or combined reactions thereof. As pyrrolic metabolites are suspected to play a crucial role in tumor initiation, our results gave a special focus on this metabolic pathway. Oxidation reactions via dehydrogenation of the necine base and to a lesser extent of the necic acid were also detected. The screening for GSH conjugates revealed the mono- and diGSH-DHP metabolites as well as newly detected conjugates. The screenings with the other trapping agents, i.e., KCN and NAC, did not reveal any further new reactive metabolites.

**Fig. 3:**
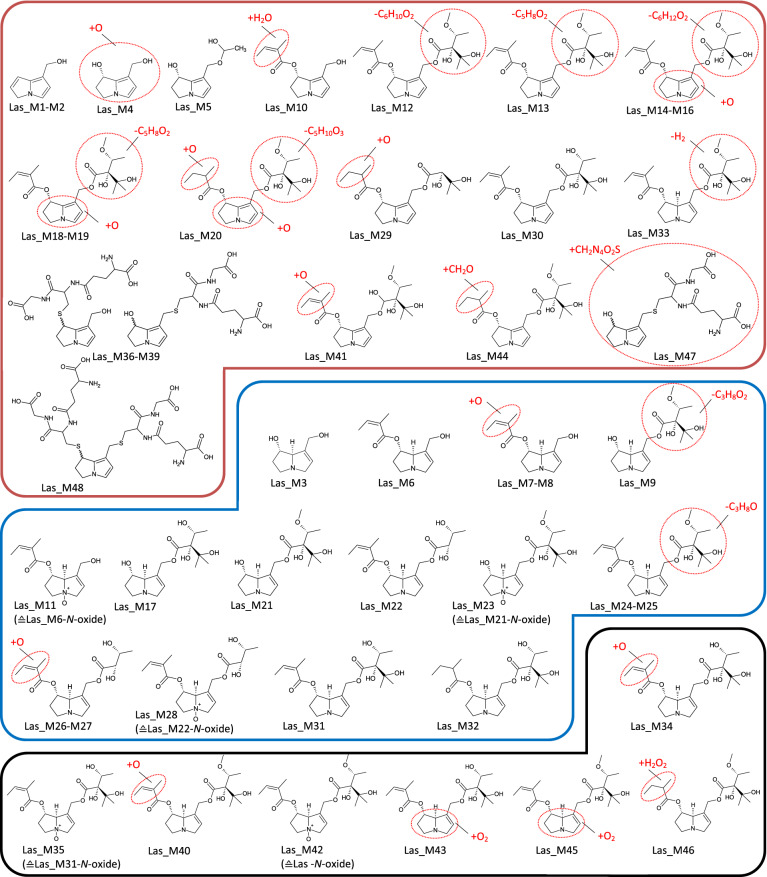
Tentative structures of lasiocarpine metabolites after incubation with human and rat liver microsomes. In some cases, biotransformation could only be assigned to distinct regions of the molecule (encircled in red). The metabolites were summarized into three groups: red box—dehydrogenation group (the insertion of an additional double bond); blue box—shortening of alkyl chain (including combinations thereof); black box—oxygenation reactions (color figure online)

**Fig. 4:**
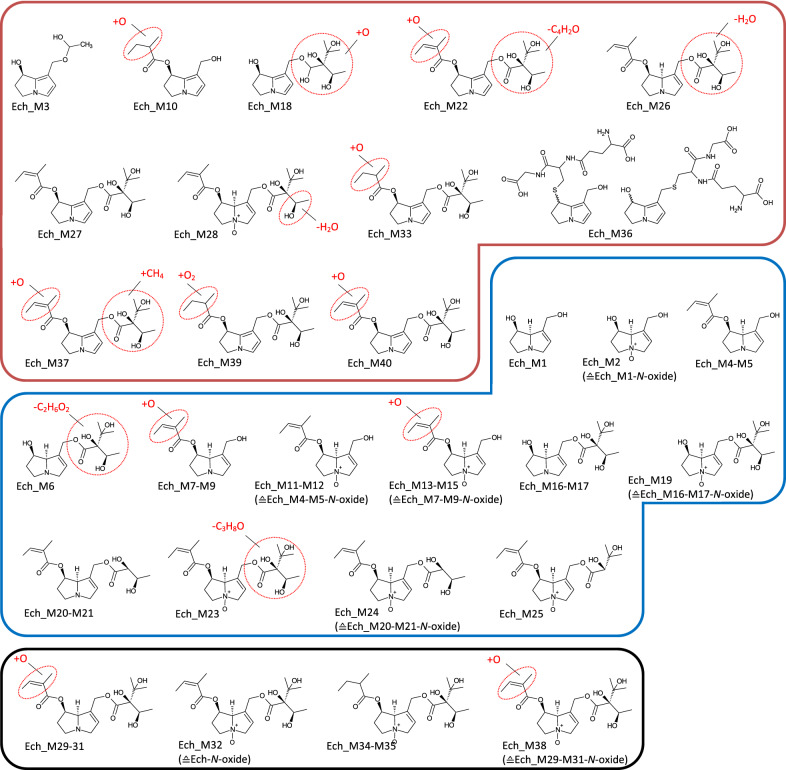
Tentative structures of echimidine metabolites after incubation with human and rat liver microsomes. In some cases, biotransformation could only be assigned to distinct regions of the molecule (encircled in red). The metabolites were summarized into three groups: red box—dehydrogenation group (the insertion of an additional double bond); blue box—shortening of alkyl chain (including combinations thereof); black box—oxygenation reactions (color figure online)

**Fig. 5:**
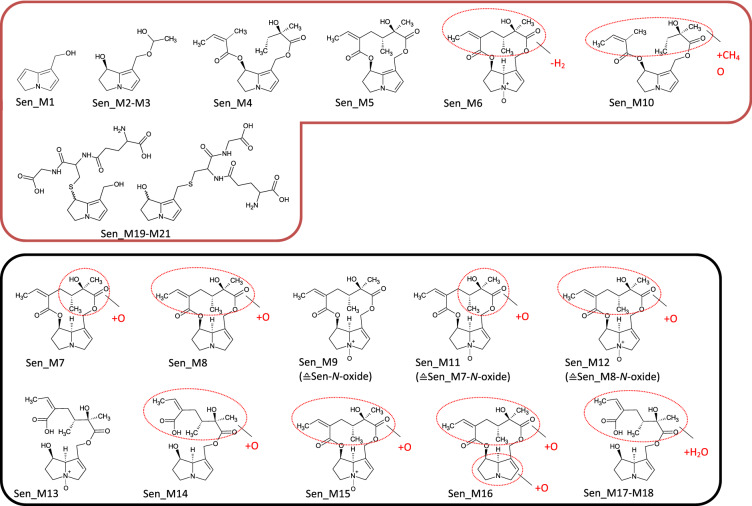
Tentative structures of senecionine metabolites after incubation with human and rat liver microsomes. In some cases, biotransformation could only be assigned to distinct regions of the molecule (encircled in red). The metabolites were summarized into two groups: red box—dehydrogenation group (the insertion of an additional double bond); black box—oxygenation reactions (color figure online)

**Fig. 6:**
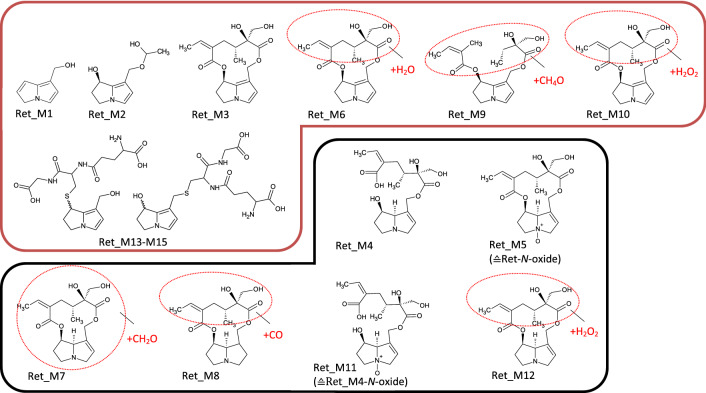
Tentative structures of retrorsine metabolites after incubation with human and rat liver microsomes. In some cases, biotransformation could only be assigned to distinct regions of the molecule (encircled in red). The metabolites were summarized into two groups: red box—dehydrogenation group (the insertion of an additional double bond); black box—oxygenation reactions (color figure online)

**Fig. 7:**
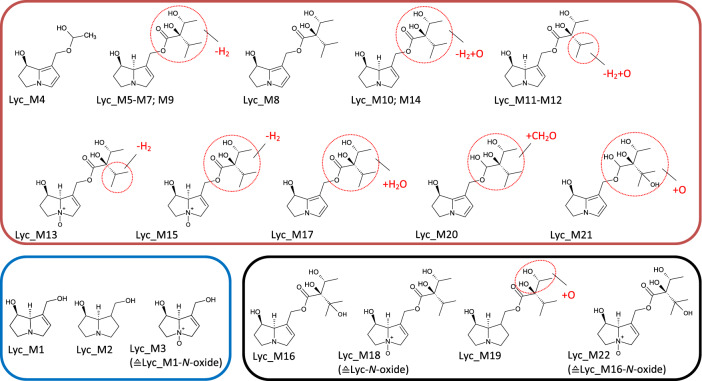
Tentative structures of lycopsamine metabolites after incubation with human and rat liver microsomes. In some cases, biotransformation could only be assigned to distinct regions of the molecule (encircled in red). The metabolites were summarized into three groups: red box—dehydrogenation group (the insertion of an additional double bond); blue box—shortening of alkyl chain (including combinations thereof); black box—oxygenation reactions (color figure online)

**Fig. 8:**
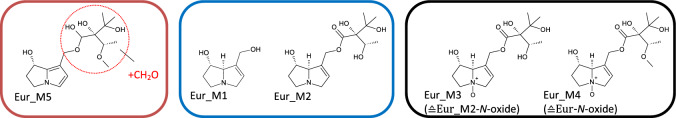
Tentative structures of europine metabolites after incubation with human and rat liver microsomes. In some cases, biotransformation could only be assigned to distinct regions of the molecule (encircled in red). The metabolites were summarized into three groups: red box—dehydrogenation group (the insertion of an additional double bond); blue box—shortening of alkyl chain (including combinations thereof); black box—oxygenation reactions (color figure online)

In Fig. 2, listed metabolites are subdivided according to their biotransformation step into transformation groups (TG). The stated transformation change in this figure represents the absolute change of the elemental composition. For instance, for Las_M32, the transformation change between the parent compound and the metabolite is a single carbon atom. Fragmentation patterns obtained by recording product ion spectra revealed this absolute elemental change to be the result of a hydrogenation of the necic acid at C7 combined with a parallel release of the methyl group from the necic acid at C9. Based on these results, the mass spectrometric fragmentation pattern of each metabolite could be assigned specifically or at least allocated to a certain part of the molecule (Figs. 3, 4, 5, 6, 7, 8).

### Lasiocarpine

Figure 2a shows an overview of the 48 identified metabolites of lasiocarpine and their time-dependent formation in rat and human liver microsomal preparations. Rapid metabolization of lasiocarpine was observed for both species. After 60 min, between 90% and more than 99% of the initial lasiocarpine amount was transformed by HLM and RLM, respectively. The proposed molecular structures of the metabolites are shown in Fig. 3. As mentioned above, the metabolites were classified into different groups according to their underlying biotransformation steps. The ‘dehydrogenation group’ (Group -H_2_) includes all metabolites with at least one additional double bond in the necine base or the necic acid (red box in Fig. 3) and accounts for 8% (RLM) or 35% (HLM), respectively, of the total metabolite peak areas. The total metabolite peak area was determined by summing all metabolite peak areas of lasiocarpine individually for each time point. Three of the dehydrogenated metabolites were additionally oxygenated within the necine base. None of these kinds of metabolites could be detected for any other investigated PA. This group also contains the dehydrogenated metabolites conjugated with GSH. As shown in Figs. 2a, 3, for lasiocarpine, 24 metabolites of those ‘pyrrolic’ metabolites could be identified, including Las_M47, which represents a new GSH conjugate not having been observed yet. The structure was confirmed by comparing product ion spectra (refer to Fig. 10 in the supplementary) with synthesized GSH-DHP conjugates showing a high degree of similarity in fragmentation. The ddMS2 data indicate that the new conjugate basically looks like the monoGSH-DHP conjugate, being modified or extended, however, by an additional alkyl chain. All other metabolites without an additional insertion of a double bond are either summarized in Group + O or Group-C. Group + O includes oxygenation products shown in the black box in Fig. 3, representing 11% (RLM) or 14% (HLM), respectively, of the total metabolite area. Group-C which dealkylated, e.g., demethylated, products are summarized in the blue box in Fig. 3 represents 80% (RLM) or 50% (HLM), respectively, of the total metabolite area. Metabolite Las_M42 represents the *N-*oxide and is formed by both species. The main metabolite in both species is the demethylation product Las_M31. In the case of RLM, the maximum concentration of Las_M31 was already reached after 10 min and its amount decreased over time. Las_M3 was identified as the necine base formed by the hydrolysis of the ester bonds at C7 and C9. However, this transformation could only be detected in RLM. Incubations of lasiocarpine were performed for both species with glutathione to simulate phase II metabolism (results are shown in Fig. 2a), and without glutathione to represent only phase I metabolism (data not shown). By adding GSH, some metabolites could not be detected anymore in supernatants of phase II preparations which suggests that GSH was acting as a trapping agent. Some of these metabolites are isomers of Las_M5, Las_M14, or Las_M18, and were only detected in low concentrations. Nevertheless, all metabolites solely formed in the absence of GSH contain an additional double bond and thus belong to the dehydrogenation group. In turn, GSH conjugates were only formed after addition of GSH. Despite the large number of identified metabolites and the nearly complete disappearance of lasiocarpine, a comparatively high fraction of metabolized lasiocarpine remained unknown and ranged between 10% (*t* = 5 min) and 70% (*t* = 60 min) for both species, meaning that 70% of the converted lasiocarpine could not be identified.

### Echimidine

In total, 40 metabolites were identified from echimidine, as shown in (Fig. 2b). After 60 min, 20% of the parent PA were metabolized by HLM, while 70% were metabolized by RLM. Detected metabolites were grouped according to the same criteria as described for lasiocarpine. Metabolization through ester cleavage and loss of alkyl groups (Group-C) was the predominant metabolic pathway (50% in RLM and 61% in HLM). Both the main metabolite in HLM (Ech_M4) and RLM (Ech_M17) represent ester cleavages at C9 and C7, respectively, accounting for 33% and 38% of the total metabolite area, and can thus be attributed to this group. For echimidine, the formation of the necine base by cleavage of both ester bonds (Ech_M1: retronecine) could be detected for both species. Echimidine formed ten dehydrogenated metabolites (Group-H_2_) representing 10% of the total metabolite area in RLM, and eight in HLM (up to 25% of the total metabolite area). For most of these dehydrogenated metabolites, the double bond is located within the necine base, except for Ech_M26 and Ech_M28. Only one GSH conjugate, namely monoGSH-DHP, could be detected (Ech_M36). The group of oxygenation products (Group + O; Ech_M29-M32, Ech_M34-M35; Ech_M38) represented a relative amount between 16% (HLM) and 40% (RLM) of the total metabolite area. The main metabolite in this group was the *N*-oxide (Ech_M32), representing up to 18% of the total metabolites. Three metabolites were solely detectable under phase I conditions and seem to be trapped by the addition of GSH. Comparable to lasiocarpine, those metabolites mainly belonged to the dehydrogenation group, while one metabolite was an oxygenation product. All of them were detectable in trace amounts and do not affect the quantitative balance of the metabolism. 10–30% of the metabolized PA remained unknown.

### Senecionine

Figures 2c and 5 show the 21 identified metabolites generated by incubation of senecionine with HLM and RLM. While RLM almost fully transformed the parent PA, about 70% remained unmetabolized in HLM. For senecionine ten dehydrogenation products could be identified. For nine of these metabolites, the additional double bond was located in the necine base, for one metabolite (Sen_M6) in the necic acid part. This metabolite group (Group-H_2_) included the GSH-DHP conjugates and represented 1% (RLM) or 20% (HLM) of the total metabolite area. Senecionine *N*-oxide (Sen_M9) was the main metabolite in both species accounting for 60% in HLM and 95% in RLM. Generally, oxygenation reactions (Sen_M7–M9, Sen_M11–M18) represented 80% (HLM) and 99% (RLM) of all metabolites. The non-identified portion was about 25% after 60 min in microsomes from both species. Eight metabolites were solely detectable under phase I conditions, i.e., could not be determined in the presence of glutathione. Two of them contained an additional double bond in their structure, while six metabolites were oxygenation products.

### Retrorsine

In total, 15 different metabolites were identified from retrorsine (Figs. 2d, 6), a number comparable to senecionine. At the end of the incubation period, about 20% of the PA was transformed by HLM and about 55% by RLM. The major proportion was oxygenation products (Group + O) accounting for about 80–90% of the total metabolite area for both species. Six metabolites could be assigned to this group (Ret_M4–M5; Ret_M7–M8; Ret_M11–M12). As observed for senecionine, the *N* oxide (Ret_M5) was the main metabolite accounting for about 75% (HLM) and 90% (RLM) of the total metabolite area. Nine metabolites dehydrogenated within the necine base were detected (Group-H_2_) and accounted for 3% (RLM) and 19% (HLM) of all metabolites. The unknown fraction for both species increased with prolonged incubation times from 5% after 5 min to 15% after 60 min. Four of the five metabolites formed solely under phase I conditions possessed an additional double in the necine base and accounted for up to 5% in RLM and 20% in HLM of the total metabolite profile of phase I.

### Lycopsamine

Twenty-second different metabolites were identified from the monoester lycopsamine, as shown in Figs. 2e, 7. Most notably, in contrast to diester PAs, almost no transformation took place by HLM. In RLM, a number of metabolites were identified such as Lyc_M1–M3, i.e., products of C9 ester cleavage including the free retronecine base, and both, in the *N*-oxidized and the 1,2-saturated form, too (Group-C). They accounted for about 4% of the total metabolites. For lycopsamine, 15 different metabolites were identified and could be assigned to the dehydrogenation group representing 6% of the total metabolite area. Ten of these metabolites contained an additional double bond in the necic acid (Lyc_M5–M7; Lyc_M9–M15) and represented this group almost entirely. Five of these metabolites, found in trace amounts only, were dehydrogenated in the necine base. Lyc_M4, which belongs to this group, was the only lycopsamine metabolite identified in HLM. Oxygenation of the necic acid was the main metabolic pathway and represented 90% of the total metabolite profile at all time points (Lyc_M16, Lyc_M18–M19; Lyc_M22). Lyc_M16 was the main metabolite in RLM (89%). Monoesters like lycopsamine differed from diester PAs as their metabolite profile was not affected by the addition of GSH. The same metabolites were detectable in phase I as well as in phase II. No metabolites seemed to be trapped by GSH and no GSH conjugates were detected either. The unknown fraction was rather small and not exceeding 17% (RLM, t60 min).

### Europine

For europine, a comparatively low number of five metabolites was determined (Figs. 2f, 8) due to the fact that even after 60 min of incubation with RLM, more than 80% of the parent PA was still present. As already published for other monoesters like lycopsamine, europine was hardly metabolized by HLM (Geburek et al. 2020). Metabolization to the heliotridine necine base (Eur_M1) and demethylation of the methoxy-group (Eur_M2) finally resulted in metabolites with an alkyl chain shorting of the necic acid (Group-C). Similar to lasiocarpine, this metabolite (Eur_M2) was the main product accounting for 93% of the total metabolite area. Europine *N-*oxide (Eur_M4) and the *N*-oxide of Eur_M2 (Eur_M3) were oxygenation products with europine *N-*oxide being the second most prominent of all europine metabolites (6%). Both of these *N *oxides were the only europine metabolites found in HLM. In RLM, europine was transformed into one dehydrogenation product only (Eur_M5) accounting for about 0.2% of the total metabolite profile. When comparing phase I and II metabolism, no change in the metabolite profile was observed. As for lycopsamine, no GSH conjugates were detected. The metabolite profile of europine could be identified almost completely and comprised of about 1% the lowest unknown fraction of all PAs.

## Discussion

The results presented in this paper indicate that human as wells as rat liver microsomes convert PAs into a much higher number of different metabolites than previously described (Buhler and Kedzierski 1986; Chung and Buhler 1994; Couet et al. 1996; Fu et al. 2004b; Mattocks and Bird 1983; Reed et al. 1992; Samuel and Jago 1975). Taken together, over 150 metabolites of six different PAs were detected. Quantification of the metabolites showed a significant decline in the unknown fraction of the transformed PA. For example, with all previously known metabolites for lycopsamine, 95% of the transformed PA would remain unknown, while the additionally identified metabolites decreased the unknown proportion to 17%. The software-assisted metabolomic workflow allowed recognizing chromatographic peaks present in the incubation samples but absent in the blanks or controls. This search was applied with a minimum of limitations concerning the peak recognition algorithm and the results were sorted with wide-set filters. The reliability of unknown workflow results was verified by checking whether all already known metabolites were fully detected in this approach. Since this requirement was fulfilled and a considerable number of hitherto unknown metabolites could be identified, it can be assumed that the remaining unknown fraction does not consist of further unknown water-soluble metabolites, except very small molecules of high polarity which could not be detected with the applied methods. Rather, it could be a result of irreversibly tissue-bound metabolites precipitated within the protein fraction and, therefore, not present in the supernatant and, consequently, not accessible to direct MS detection. The highest unknown fraction was observed for lasiocarpine (70%, *t* = 60 min, HLM and RLM) and the lowest for europine (1%, *t* = 60 min, HLM and RLM). This difference supports the assumption that lasiocarpine, one of the most toxic PA (Efsa 2011; NTP 1978; Stegelmeier et al. 2016), forms much more metabolites irreversibly binding to proteins than europine.

**Fig. 9:**
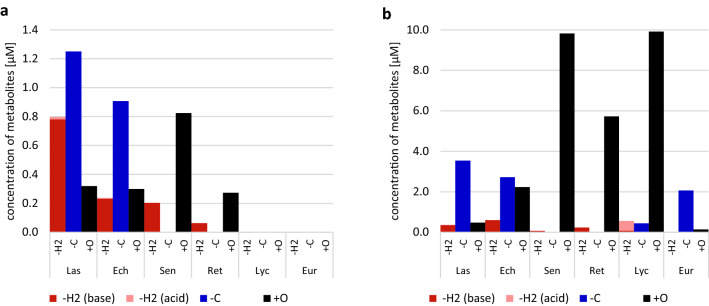
Grouped metabolite concentration according to sub-groups for each PA (initially 15 µM) either in human (**a**, left) or rat liver microsomes (**b**, right): –H2: dehydrogenated metabolites (insertion of an additional double bond either in necine base, dark red, or in the necic acid, light red); –C: shortening of alkyl chain. + O: oxygenation products, including combinations thereof (color figure online)

As already demonstrated in earlier studies, a major metabolic pathway for open chained diesters is shortening of necic acids by demethylation and dealkylation. Finally, the cleavage of both ester bonds results in the necine base retronecine or heliotridine, respectively (Fashe et al. 2015; Kedzierski and Buhler 1985; Mattocks 1968; McLean 1970; Samuel and Jago 1975). This metabolic pathway is generally regarded as detoxification and comprises the largest proportion of metabolites (Fig. 9, blue bars). The formation of *N *oxides was also described as detoxification step, but was only detected in minor amounts for open chained diesters, either because *N *oxides were not formed or because they were rapidly metabolized further.

The oxidation of the heterocyclic carbon by dehydrogenation is generally considered as the major metabolic step to toxicity (Fu et al. 2004a) and was suggested to play an important role in the initiation of PA-induced liver tumors (Xia et al. 2018). In this study, lasiocarpine by far led to the highest amount of dehydrogenated metabolites of this type in both species (Fig. 9, dark red bars). In addition to ‘pyrrolic’ metabolites formed in the phase II approach, a few metabolites were identified which were solely detectable in the phase I approach without GSH. For lasiocarpine, seven metabolites with an additional double bond in the necine base could be detected, while two were found for echimidine. In the present experimental setup, only the phase II approach was fortified with GSH and reactive phase I metabolites are expected to be trapped as GSH conjugates. Surprisingly, no direct conjugation of primary ‘pyrrolic’ metabolites with GSH but only the formation of mono- and diGSH-DHP conjugates was found. Apparently, ‘pyrrolic’ metabolites, other than GSH conjugates, occur in the absence of GSH which is in accordance with the findings by Fashe et al. (Fashe et al. 2015). Only a few metabolites could be detected that were commonly formed by the open chained diesters echimidine and lasiocarpine (compare m/z 156, m/z 198, m/z 238, m/z 254) or represent the same transformation change, for example −C_5_H_6_O, + O or + CH_2_O. These findings illustrate that fundamental differences in the type and intensity of certain metabolic steps may occur between structurally related PA congeners.

Contrary to the open chained diesters, the main metabolic pathway of cyclic diesters can be assigned to oxygenation reactions (black bars in Fig. 9). The *N* oxides of retrorsine and senecionine were the main metabolites and presented on average a proportion of 95% of all metabolites. Furthermore, the introduction of a single or two oxygen atoms within the necic acid could be detected and up to 11 respective metabolites were identified. As already described (Mattocks 1986), transformation products resulting in an opening of the macrocyclic ring were detected (Figs. 5, 6). Retronecine as final cleavage product could not be detected, however. Ten metabolites were commonly formed by both cyclic diesters (compare, e.g., m/z 136, m/z 198, and m/z 368). Between nine and ten dehydrogenation products, most of them with an additional double bond in the necine base could be detected and quantified, and accounted for 1% (RLM) and 20% (HLM) of all formed metabolites (red bars in Fig. 9). The results of our study are in agreement with the previous investigations for cyclic diesters (Xia et al. 2020) showing *N*-oxide formation and other oxygenation steps as main metabolic routes (Xiong et al. 2012). Furthermore, DHP and GSH-DHP conjugates were reported to be formed in relevant amounts, while the determination of dehydrogenated PAs was not possible as they were assumed to be instable (Huan et al. 1998a; Kedzierski and Buhler 1986; Miranda et al. 1991; Ramsdell and Buhler 1987; Reed et al. 1992; Styles et al. 1980; Wang et al. 2005). In our study, these dehydrogenated PAs and GSH-DHP conjugates were formed in higher quantities, but only traces of DHP were detectable (DHP amounts were below the limit of quantification and,, therefore are not shown in Fig. 2).

Earlier studies from our laboratory showed that monoesters are not or only marginally metabolized by human liver microsomes. The low transformation of monoesters by HLM seems to be due to the free alcoholic group at the C7 position of the PA molecules, as it has been shown that acetylation of this position is sufficient to achieve almost complete metabolic transformation (Geburek et al. 2020). In contrast, incubation with rat liver microsomes resulted in an almost complete transformation, at least for lycopsamine and to 20% for europine. Species-specific differences in metabolic pathways may result from differences in gene expression and/or function of xenobiotic-metabolizing enzymes (Martignoni et al. 2006). Similar to open chained diesters, almost no *N*-oxide formation from monoesters was detected. Lycopsamine formed many metabolites with an additional double bond in the necic acid, which were also summarized as dehydrogenation products, but notably almost no metabolites being dehydrogenated in the necine base were found (Fig. 9). Furthermore, no formation of GSH conjugates was observed either.

In contrast, metabolites dehydrogenated in the necine base and GSH conjugates thereof were detected for all diesters with the highest levels being observed for lasiocarpine (Fig. 9). Interestingly, five lasiocarpine metabolites with a dehydrogenated and oxygenated necine base were formed. These results explain the finding that the monoesters europine or lycopsamine form less DNA-reactive intermediates and lower DNA adduct levels, e.g., in rat hepatocytes than lasiocarpine or echimidine (Allemang et al. 2018; Lester et al. 2019; Louisse et al. 2019). Similarly, Gao et al. also reported much lower cytotoxicity-related EC_50_ values in primary rat hepatocytes for the diesters lasiocarpine, echimidine, senecionine, and retrorsine in primary rat hepatocytes than for europine and lycopsamine (Gao et al. 2020). Another major difference between the monoesters and diesters is the proportion of the unknown fraction, accounting for up to 70% in the latter but only 17% in the former. This finding suggests that monoesters, in contrast to diesters, form metabolites which bind to tissue constituents to a lesser extent.

## Conclusions

In the present study, we used human and rat liver microsomal preparations to metabolize six structurally different PAs and analyzed the metabolite profiles by mass spectrometric methods. These results are an important mosaic stone in the overall pattern of PA toxicokinetics needed for a refined risk assessment of PA exposure. Although the major correlations between structures and activation vs. detoxification pathways were confirmed in our study, more data on uptake, transport, and disposition of PAs are needed to obtain a more complete picture. Between PAs and species similarities but also marked differences in metabolism were found, indicating that results from rat studies with PAs should not be considered to mirror the human situation by default. Although the number of detectable metabolites correlated with the depletion of the parent PA, the highest number with 40–48 different metabolites was identified for open chained diesters. For cyclic diesters, about 15–20 metabolites were detected and 22 were found for the monoester lycopsamine, while only five metabolites were identified for europine. Beside the fact that the free alcoholic group at C7 seems to prevent monoester from metabolic transformation, we previously described that the overall degradation of PAs seems to correlated with their polarity, whereby the more polar and branched-chained PAs exhibited lower degradation. Consequently, the comparatively high lipophilicity of open chained diesters favors their metabolism in general. Combined with the higher number of carbons that are attackable for metabolic transformation, the difference in the number of metabolites for the different PAs could be explained. In the case of the open chained diesters, the main metabolic pathway can be summarized as necic acid shortening including demethylation and loss of larger alkyl groups, whereas the cyclic diesters mainly underwent oxygenation reactions, with the formation of the *N* oxide as the main pathway. The monoester lycopsamine was mainly metabolized by oxygenation and dehydrogenation of the necic acid moiety. For half of the metabolites identified in this study, a structural change in the necine base by the formation of a further double bond could be observed, a step considered as bioactivation (Fu et al. 2004b; Ruan et al. 2014b). While almost none of these dehydrogenated metabolites were detected in incubations of monoesters, all diesters investigated were metabolized to such products. The amounts detected, with lasiocarpine forming the highest number and amount of dehydrogenation products, were in good correlation to the toxicity described for individual congeners. Furthermore, GSH conjugates were detected for both the open chained and cyclic diesters, which is an indirect evidence for the formation of reactive metabolites. Although metabolic pathways of PAs are generally similar, only a very limited number of commonly formed metabolites, such as mono-DHP-GSH, could be detected. This limits the identification of a generic biomarker for PA exposure. Therefore, it is important to study the significance of the metabolites in terms of toxicity, as a biomarker for toxic PAs is more important than a biomarker for general PA exposure.

## Electronic supplementary material

Below is the link to the electronic supplementary material.

Supplementary file1 (DOCX 177 kb)

## Data Availability

Not applicable.
