# Supplementary material for: In vitro biotransformation of pyrrolizidine alkaloids in different species: part II—identification and quantitative assessment of the metabolite profile of six structurally different pyrrolizidine alkaloids
Source: Arch Toxicol. 2020 Sep 3;94(11):3759–74. doi: 10.1007/s00204-020-02853-9 (PMC7603446; doi:10.1007/s00204-020-02853-9)
Supplement: Supplementary file 1 — Supplementary file1 (DOCX 177 kb) [file 204_2020_2853_MOESM1_ESM.docx]

**Supplementary Material**


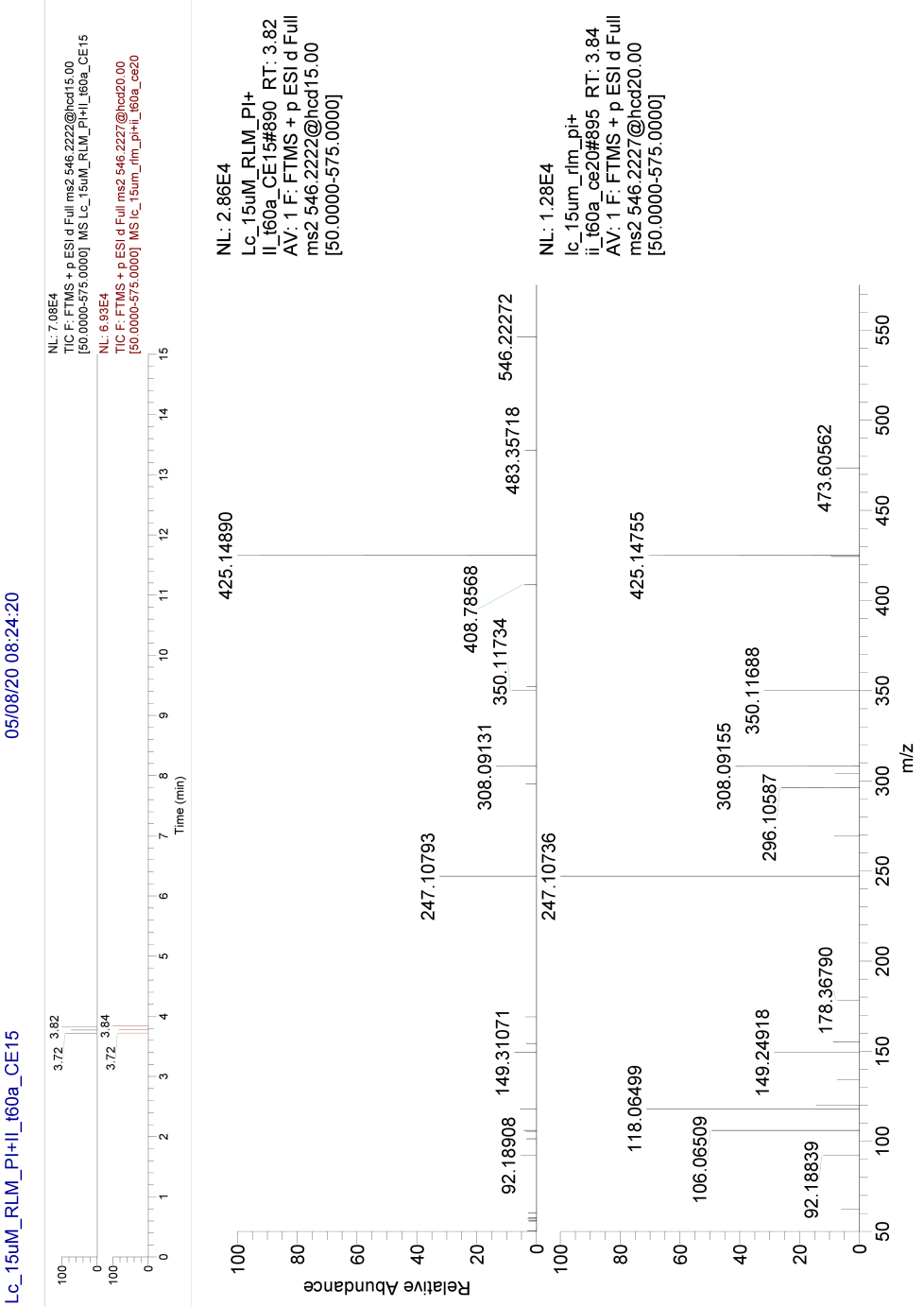


**Fig. 10** Product ion spectra of the metabolite Las_M47 (C22H35N5O6S, m/z 546.22283), a new GSH conjugate, detected by tandem mass spectrometry and collision energy of 15 eV and 20 eV.
